# Uncovering allosteric communication in cancer-related histone mutations

**DOI:** 10.1093/bib/bbaf631.022

**Published:** 2025-12-12

**Authors:** Shuxiang Li, Jie Shu, James C Rober, Austin Macklem, Daniel Espiritu, Tanay Debnath, Samuel Tian, Daniel Tian, Maria J Aristizabal, Anna R Panchenko

**Affiliations:** Department of Pathology and Molecular Medicine, Queen’s University, ON, Canada; Biology Department, Queen’s University, ON, Canada; Department of Biomedical and Molecular Sciences, Queen’s University, ON, Canada; Biology Department, Queen’s University, ON, Canada; Department of Pathology and Molecular Medicine, Queen’s University, ON, Canada; Department of Pathology and Molecular Medicine, Queen’s University, ON, Canada; School of Computing, Queen’s University, ON, Canada; School of Computing, Queen’s University, ON, Canada; Biology Department, Queen’s University, ON, Canada; Department of Biomedical and Molecular Sciences, Queen’s University, ON, Canada; Department of Pathology and Molecular Medicine, Queen’s University, ON, Canada; Department of Biomedical and Molecular Sciences, Queen’s University, ON, Canada; School of Computing, Queen’s University, ON, Canada; Ontario Institute of Cancer Research, Toronto, ON, Canada; Physics Department, Queen’s University, ON, Canada

## Abstract

**Background:**

Histone mutations have been implicated in various cancers, but their mechanistic effects on chromatin dynamics remain largely unexplored.

**Aim:**

To investigate how allosteric communication influences the functional outcomes of cancer-linked histone mutations.

**Methods:**

We analyzed forty cancer-associated histone missense mutations using integrated computational modeling and experimental validation to evaluate their long-range structural and functional consequences.

**Results:**

The allosteric effects of histone mutations were highly position-dependent. Mutations near the N-terminal tails of histones H3 and H4 caused the most pronounced conformational perturbations. Seven histone mutations were predicted to have strong allosteric signatures potentially influencing nucleosome interactions. Experimental assays confirmed that H2BS64Y and H2BS64F substitutions impaired histone function, disrupted H2BK120 ubiquitination, and decreased genome stability.

**Conclusions:**

These findings indicate that certain histone mutations may exert oncogenic potential through long-range allosteric mechanisms, underscoring the importance of allostery as a key regulatory principle in cancer epigenetics.

